# Association of objectively measured physical activity and sedentary time with arterial stiffness in women with systemic lupus erythematosus with mild disease activity

**DOI:** 10.1371/journal.pone.0196111

**Published:** 2018-04-25

**Authors:** Pablo Morillas-de-Laguno, José A. Vargas-Hitos, Antonio Rosales-Castillo, Luis Manuel Sáez-Urán, Cristina Montalbán-Méndez, Blanca Gavilán-Carrera, Carmen Navarro-Mateos, Pedro Acosta-Manzano, Manuel Delgado-Fernández, José M. Sabio, Norberto Ortego-Centeno, José L. Callejas-Rubio, Alberto Soriano-Maldonado

**Affiliations:** 1 Department of Physical Education and Sport, Faculty of Sport Sciences, University of Granada, Granada, Spain; 2 Systemic Autoimmune Diseases Unit, Department of Internal Medicine, “Virgen de las Nieves” University Hospital, Granada, Spain; 3 Fellows of Systemic Autoimmune Diseases Unit, Department of Internal Medicine, “Virgen de las Nieves” University Hospital, Granada, Spain; 4 Systemic Autoimmune Diseases Unit, Department of Internal Medicine, “San Cecilio” University Hospital, Granada, Spain; 5 Department of Education, Faculty of Education Sciences, University of Almería, Almería, Spain; 6 SPORT Research Group (CTS-1024), CERNEP Research Center, University of Almería, Almería, Spain; Peking University First Hospital, CHINA

## Abstract

**Objectives:**

To examine the association of objectively measured physical activity (PA) intensity levels and sedentary time with arterial stiffness in women with systemic lupus erythematosus (SLE) with mild disease activity and to analyze whether participants meeting the international PA guidelines have lower arterial stiffness than those not meeting the PA guidelines.

**Methods:**

The study comprised 47 women with SLE (average age 41.2 [standard deviation 13.9]) years, with clinical and treatment stability during the 6 months prior to the study. PA intensity levels and sedentary time were objectively measured with triaxial accelerometry. Arterial stiffness was assessed through pulse wave velocity, evaluated by Mobil-O-Graph® 24h pulse wave analysis monitor.

**Results:**

The average time in moderate to vigorous PA in bouts of ≥10 consecutive minutes was 135.1±151.8 minutes per week. There was no association of PA intensity levels and sedentary time with arterial stiffness, either in crude analyses or after adjusting for potential confounders. Participants who met the international PA guidelines did not show lower pulse wave velocity than those not meeting them (b = -0.169; 95% CI: -0.480 to 0.143; *P* = 0.280).

**Conclusions:**

Our results suggest that PA intensity levels and sedentary time are not associated with arterial stiffness in patients with SLE. Further analyses revealed that patients with SLE meeting international PA guidelines did not present lower arterial stiffness than those not meeting the PA guidelines. Future prospective research is needed to better understand the association of PA and sedentary time with arterial stiffness in patients with SLE.

## Introduction

Systemic lupus erythematosus (SLE) is an autoimmune disease of unknown aetiology that predominantly affects young adult women. The prognosis of SLE has improved significantly in recent decades [1] and has led to new comorbidities, such as clinical and subclinical atherosclerotic cardiovascular diseases (CVD) [2]. In fact, CVD, which are characteristically of early presentation and accelerated evolution [3], have become the main cause of mortality in this population [3].

Arterial stiffness is a marker of subclinical atherosclerosis that allows the detection of mechanical changes in the distensibility of the arteries previous to atherosclerosis development, and is a powerful predictor of CVD independently of the presence of other cardiovascular risk factors [4,5]. Arterial stiffness, measured by pulse wave velocity (PWV), is significantly increased in patients with SLE [6,7] and is associated with the development of CVD [8]. Therefore, understanding potential modifiable factors that might be associated with lower arterial stiffness in patients with SLE is of clinical and public health interest.

Physical activity (PA) is defined as any bodily movement produced by skeletal muscles that results in energy expenditure. PA in daily life can be categorized into occupational, sports, conditioning, household, or other activities [9]. The term ‘sedentary’ can operationally be defined as any waking sitting or lying behaviour with low energy expenditure [10]. The term ‘sedentary behaviour’ therefore typically refers to sitting/lying behaviour rather than a simple absence of moderate-to-vigorous PA (MVPA) [10]. There is enough information about exercise physiology to support the well-documented public health guidelines promoting at least 150 min/week of moderate-vigorous leisure-time PA aimed at decreasing risks for metabolic diseases [11]. Moreover, current physical activity guidelines, including patients with rheumatic diseases, recommend a minimum of 150 minutes of MVPA per week accumulated in bouts lasting at least 10 minutes [12]. In patients with SLE, it has been observed that low PA levels were associated with increased subclinical atherosclerosis, and more severe organ damage was associated with less PA of “low to moderate” intensity, compared to general population [13,14]. In addition, it has been suggested that in adults, long lasting periods of sedentary time are associated with a worse cardiometabolic risk profile, and greater risk of clinical and subclinical atherosclerosis [15]. Furthermore, in general population, daily sitting time or low non-exercise activity levels may have a significant direct relationship with each of these medical concerns: mortality, CVD, type 2 diabetes, metabolic syndrome risk factors and obesity [11]. It has been evidenced that endothelial function declines with age and sedentary lifestyle, and this is associated with an increased risk for CVD in general population [16]. Most of previous research has been conducted in the general population or in patients with CVD. However, no prior study has examined the extent to which physical activity or sedentary time (objectively measured by accelerometry) might be associated with arterial stiffness in patients with SLE. We hypothesized that lower levels of PA and higher sedentary time would be associated with higher arterial stiffness in patients with SLE.

Therefore, the present study aimed to examine the association of objectively measured PA intensity levels and sedentary time with arterial stiffness in patients with SLE and whether participants meeting the international PA guidelines present lower arterial stiffness than those not meeting the PA guidelines.

## Materials and methods

### Study design and participants

In this cross-sectional study, a total of 144 women with SLE were invited to participate in the study from the Systemic Autoimmune Diseases Unit of the “Virgen de las Nieves” University Hospital or the “San Cecilio” University Hospital.

Inclusion criteria were: fulfilling ≥ 4 ACR classification criteria [17], at least 1 year of follow-up at the respective autoimmune unit and presenting clinical stability (defined as no changes in Systemic Lupus Erythematosus Disease Activity Index (SLEDAI) and/or the treatment) during the 6 months prior to the beginning of the study. Exclusion criteria were: not being able to read, understand and/or sign the informed consent, personal history of CVD in the previous year and having received a biological treatment or the need of prednisone (or equivalent) higher than 10 mg / day in the past 6 months. Furthermore, in the accelerometry analysis, a minimum of ten or more hours of registration per day during the seven days of recording was necessary to be included in the study [18].

The patients signed written informed consent after receiving detailed information about the study procedures. The study protocol was reviewed and approved by the Research Ethics Committee of Granada.

### Procedures

Each patient received a dossier with detailed information about the study design and evaluation protocols, as well as the objectives and purpose of the study.

The patients were evaluated in October 2016, where sociodemographic data and clinical history were recorded, anthropometric measures and body composition were assessed, and PWV was measured. Finally, an accelerometer was given to each participant. They were asked to wear the accelerometers for the following 9 days, and were instructed on how to fill out the sleep diary.

### Measurements

#### Sociodemographic variables and clinical history

Age, educational level, occupational status, family history of CVD, personal history of cardiovascular risk factors and treatments, and SLE data (diagnostic criteria, year of diagnosis, time of evolution and treatments) were registered by questionnaires. The disease activity was measured by the SLEDAI [19]. Accumulated organ damage was assessed by the Damage Index for Systemic Lupus Erythematosus (SDI) scale [20].

#### Anthropometric measures and body composition

Weight was measured in kilograms (InBody R20, Biospace, Seoul, Korea) and height in centimeters using a stadiometer (SECA 222, Hamburg, Germany). Body mass index (BMI; kg/m^2^) was calculated. Body fat percentage and muscle mass was measured by Bioelectrical impedance (InBody R20, Biospace, Seoul, Korea). Waist circumference (at the umbilical level) and hip circumference (at the level of the iliac crest) were measured by non-elastic anthropometric tape (SECA 200).

#### Pulse wave velocity

The Mobil-O-Graph® 24h pulse wave monitor (IEM GmbH, Stolberg, Germany), which is based on the oscillometry recorded by a blood pressure cuff placed on the brachial artery [21], was used to measure PWV. Data obtained by the recorder can be easily transferred to a computer-based central database by use of a Blue-tooth interface. The central database is a hypertension management software (IEM GmbH, Stolberg, Germany) [22]. This device has been largely shown to be valid a reliable for measuring PWV in different populations [23,24], met the accuracy requirements of the British Hypertension Society (BHS) [25–27] standard, and can be recommended for clinical use [22].

#### PA levels and sedentary time

PA was objectively measured by accelerometry [28]. Data was collected using triaxial accelerometer GT3X+ (Actigraph, Pensacola, Florida, USA), stored at an epoch length of 60 seconds and with a frequency rate of 30 Hz [29]. Participants wore the accelerometer on the hip secured with an elastic tape. The device was carried over the whole day for seven consecutive days except when bathing, swimming or sleeping, because non-sleep patterns were measured. PA was recorded up to seven days, starting from the day the participants received the accelerometers until the day that they were instructed to return the device. It was necessary a minimum of ten or more hours of registration per day during the seven days of recording to be included in the study [18]. It was considered as non-wearing time and consequently excluded from the analysis bouts of 90 continuous minutes (30 minutes small window length and 2 minutes skip tolerance) of 0 activity intensity counts [30]. Furthermore, values with recording of more than 20000 counts per minute were excluded from the analyses (potential malfunction). Accelerometer wearing time was calculated by subtracting the non-wear time and the sleeping time (obtained from the sleep diary where patients wrote time they went to bed and time they woke up) from the total registered time for each day. Sedentary time was estimated as the amount of time accumulated below 200 counts per minute (cpm) in the PA vector magnitude during periods of wear time [31]. PA intensity levels (light, moderate and vigorous) were calculated based upon recommended PA vector magnitude cut points [29–31]: 200–2689, 2690–6166 and ≥6167cpm, respectively. All values were expressed in min/day. Moderate-to-vigorous PA (MVPA) intensity level was obtained through the sum of moderate and vigorous PA. The average time per week of MVPA in bouts of ≥10 minutes (bouted MVPA) was calculated (up to 2 minutes below the cut point allowance) according to the PA recommendations for adults [12]. However, recent evidence also supports health benefits with MVPA performed in bouts shorter than 10 min [32]. It was required the ActiGraph software (Actilife version 6.11.9) for data download, reduction, cleaning and analyses.

### Statistical analysis

Descriptive statistics are provided as mean and standard deviation for quantitative variables and percentages for categorical variables. Normality of the main variables was checked using histograms and the Shapiro-Wilk test. The association of PA intensity levels (light PA, moderate PA, MVPA, total PA and bouted MVPA) and sedentary time with arterial stiffness was analyzed using linear regression models, with PWV as dependent variable and PA levels and sedentary time as independent variables in separate models. Three adjustment models were conducted: model 1 was adjusted for accelerometer-wear time; model 2 added BMI, smoking habit, blood pressure to model 1; model 3 added age to model 2. Further adjusting for SLEDAI did not change the coefficients. Normality of the residuals was analyzed and was reasonably met. Differences in PWV of participants meeting vs. not meeting the PA guidelines were assessed with analysis of covariance (ANCOVA), where PWV was entered as dependent variable, the group (0 = not meeting the guidelines; 1 = meeting the guidelines) was entered as independent variable, and accelerometer-wear time, BMI, smoking habit, blood pressure and age were entered as covariates. All statistical analyses were performed using the Statistical Package for the Social Sciences, version 23.0 for Windows (SPSS, IBM, Armonk, NY, USA), and statistical significance was set at *P*<0.05.

## Results

**Fig 1:**
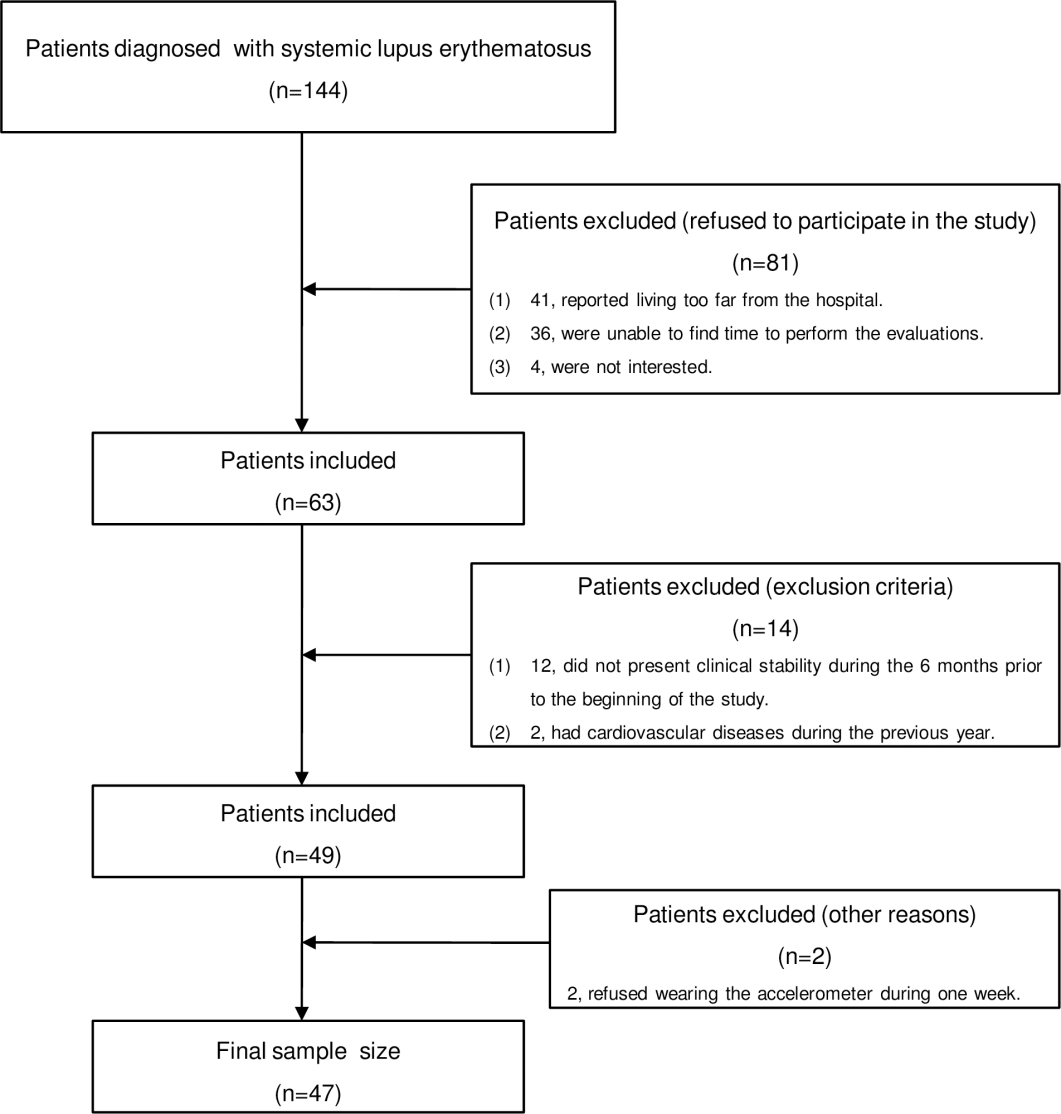
The flowchart of the selection of the patients with systemic lupus erythematosus with mild disease activity (*n = 47*).

The flowchart of the participants included in this cross-sectional study is presented in Fig 1.

Of the 144 patients initially invited, 81 refused to participate (i.e. 41 patients reported living too far from the hospital; 36 were unable to find time to perform the evaluations; and 4 were not interested), 12 patients did not present clinical stability during the 6 months prior to the beginning of the study, and 2 patients had CVD during the previous year. A total of 49 participants were finally included in the study. However, 2 patients refused wearing the accelerometer during one week. Thus, the final sample size for this study was 47 women with SLE.

The sociodemographic and clinical characteristics of the study participants are presented in Table 1. The quantitative variables were presented as mean (standard deviation; SD) and the qualitative variables as frequency and percentage (n, %). Participants were, on average, 41 years old, and 45% of participants reported an educational level of no studies or secondary school. Study participants presented a SLEDAI of 1.9 (SD 3.7) and a SDI of 0.5 (SD 0.9), and a total criteria for SLE of 5 (SD 0.8). The majority of participants (96%) were consuming hydroxychloroquine. Participants meeting the PA guidelines presented a higher percentage of renal (57.1%) and hematologic (57.1%) disease than participants not meeting the PA guidelines (30.3% and 36.4%, respectively), although these differences were not significant.

**Table 1:** Socio-demographic and clinical characteristics in women with systemic lupus erythematosus *(n = 47)*.

Characteristics	Total *(n = 47)*		Meeting PA guidelines *(n = 14)*		Not meeting PA guidelines *(n = 33)*	
Age (years; mean, SD)	41.2	13.9	46.1	12.2	39.1	14.2
Educational level (n, %)						
No education	4	8.2	3	21.4	1	3
Primary and secondary education	19	36.8	6	42.8	13	39.4
Higher education	24	55.1	5	35.7	19	57.6
Duration of SLE (years; mean, SD)	11.7	9.3	15.9	8.2	10.2	9.4
Criteria for SLE (n, % yes)						
Erythema	22	46.9	5	35.7	17	51.5
Discoid lupus	3	6.1	1	7.1	2	6.1
Photosensitivity	15	32.2	4	28.6	10	30.3
Oral ulcer	14	30.1	4	28.6	9	27.3
Arthritis	31	63.3	9	64.3	20	60.6
Serositis	15	32.2	4	28.6	10	30.3
Renal	19	40.6	8	57.1	10	30.3
Neurological	4	8.2	1	7.1	3	9.1
Hematological	21	44.8	8	57.1	12	36.4
DNA	43	87.8	11	78.6	30	90.9
ANA	46	98	13	92.9	33	100
Total criteria for SLE (mean, SD)	5	0.8	5.1	0.1	4.9	0.8
SLEDAI (mean, SD)	1.9	3.7	1.6	1.9	0.9	1.7
SDI (mean, SD)	0.5	0.9	0.3	0.5	0.6	1
Corticorteroids consumption (n, % yes)	33	70.2	9	64.3	24	72.7
Cumulative corticorteroids exposure, mg (mean, SD)	2864	2696	3131	3244	2752	2477
Drugs consumption (n, % yes)						
Hydroxychloroquine	45	95.9	13	92.9	30	90.9
Immunosuppressants (current)	21	44.8	8	57.1	13	39.4
Immunosuppressants (previous)	20	42.7	7	50	13	39.4
Antihypertensive drugs	15	32.2	5	35.7	10	30.3
Statins	8	16.4	5	35.7	3	9.1

SD: Standard Deviation; PA: Physical Activity; SLE: Systemic Lupus Erythematosus; DNA: Double Native Antibodies; ANA: AntiNuclear Antibodies; SLEDAI: Systemic Lupus Erythematosus Disease Activity Index; SDI: Damage Index for Systemic Lupus Erythematosus.

The PA intensity levels and sedentary time in women with SLE are shown in Table 2. The average accelerometer-wear time result of the included participants was 928.2 (SD 67.2) min/day. Participants showed sedentary time and light PA of 451.4 (SD 104.7) and 425.7 (SD 96.5) min/day, respectively. Furthermore, participants showed MVPA (min/day) and bouted MVPA (min/week) of 51.1 (SD 31.3) and 135.1 (SD 151.8), respectively. Besides, participants meeting the PA guidelines performed 221 min/week of bouted MVPA more than participants not meeting the PA guidelines.

**Table 2:** Physical activity intensity levels and sedentary time in women with systemic lupus erythematosus *(n = 47)*.

Variables	Total *(n = 47)*		Meeting PA guidelines *(n = 14)*		Not meeting PA guidelines *(n = 33)*	
PA and sedentary time (min/day)	Mean	SD	Mean	SD	Mean	SD
Accelerometer-wear time	928.2	67.2	946.7	64.3	920.4	67.8
Sedentary Time	451.4	104.7	392.5	111.8	476.4	92.3
Light PA	425.7	96.5	472.7	106.7	405.8	85.1
Moderate PA	48.5	29.5	75.8	36.7	36.9	16.7
Vigorous PA	2.5	6.9	5.3	11.7	1.3	3
MVPA	51.1	31.3	81.6	35.6	38.2	17.7
Bouted MVPA (min/week)	135.1	151.8	289.9	197.8	69.4	48.1

SD: Standard Deviation; PA: Physical Activity; MVPA: Moderate-to-Vigorous Physical Activity.

The CVD risk factors in women of the study are presented in Table 3. 28% of participants reported being current smokers and 16.4% of the study participants had arterial hypertension. Furthermore, study participants presented a BMI of 26.1 (SD 5.1) kg/m^2^ and a PWV of 6.2 (SD 1.4) m/s. Participants meeting the PA guidelines presented a lower BMI (24.5 kg/m^2^) and body fat (32.9%) than participants not meeting the PA guidelines (26.8 kg/m^2^ and 38.1%, respectively). Women meeting the PA guidelines presented a higher proportion of dyslipemia than those not meeting them (*P* = 0.026).

**Table 3:** Cardiovascular risk factors in women with systemic lupus erythematosus *(n = 47)*.

Variables	Total *(n = 47)*		Meeting PA guidelines *(n = 14)*		Not meeting PA guidelines *(n = 33)*		*P*
Weight (kg; mean, SD)	66.4	12.7	61.1	6	68.5	14.4	0.071
Body fat (%; mean, SD)	36.5	8.6	32.9	9.4	38.1	8.2	0.066
Waist circumference (cm; mean, SD)	83.6	10.1	80.8	8.8	84.5	11.6	0.284
Hip circumference (cm; mean, SD)	100.9	9.9	97.5	6.8	102.5	10.8	0.118
Waist/Hip ratio (mean, SD)	0.8	0.06	0.8	0.07	0.8	0.06	0.799
BMI (Kg/m^2^; mean, SD)	26.1	5.1	24.5	3.2	26.8	5.8	0.184
Tobacco consumption (n, % yes)	13	28	3	21.4	10	30.3	0.115
HTA (n, % yes)	8	16.4	2	14.3	6	18.2	0.752
Dyslipemia (n, % yes)	8	16.4	5	35.7	3	9.1	**0.026**
Diabetes (n, % yes)	1	2	0	0	1	3	0.521
Obesity (n, % yes)	6	12.2	1	7.1	5	15.2	0.463
Pulse wave velocity (m/s; mean, SD)	6.2	1.4	6.5	1.2	6.1	1.5	0.421

SD: Standard Deviation; BMI: Body Mass Index; HTA: Hypertension.

The association of PA intensity levels and sedentary time with PWV is shown in Table 4. There was no association of PA intensity levels and sedentary time with arterial stiffness, either in model 1 or after controlling for additional potential confounders (i.e. models 2 and 3). There was no bouted MVPA x age interaction on PWV (b = -0.00001; 95% CI: -0.00003 to 0.00001; *P* = 0.263). Further adjustment for SLEDAI, SDI and DMARD (hydroxychloroquine and immunosuppressants) did not modify the results and (with the aim to provide parsimonious models) we did not include these variables in the final model. As a higher proportion of physically active patients presented dyslipemia, we also included dyslipemia as a covariate but the results were unaltered.

**Table 4:** Linear regression models examining the association of physical activity intensity levels and sedentary time with pulse wave velocity in women with systemic lupus erythematosus *(n = 47)*.

Pulse wave velocity	β*	b*	(95% CI)		*P*^1^	*P*^2^	*P*^3^
Sedentary time	0.034	0.001	(-0.001,	0.002)	0.597	0.367	0.484
Light PA	-0.014	-0.001	(-0.002,	0.001)	0.467	0.290	0.776
Moderate PA	-0.067	-0.003	(-0.008,	0.001)	0.639	0.757	0.164
MVPA	-0.072	-0.003	(-0.008,	0.001)	0.683	0.879	0.132
Total PA	-0.036	-0.001	(-0.002,	0.001)	0.597	0.367	0.484
Bouted MVPA	-0.051	-0.001	(-0.001,	0.0004)	0.474	0.343	0.291

β: standardized regression coefficient; b: non-standardized regression coefficient; CI: Confidence Interval; PA: Physical Activity; MVPA: Moderate-to-Vigorous Physical Activity.

β*, b*: these values correspond to the complete model (model 3).

^1^Model 1: adjusted by accelerometer-wear time.

^2^Model 2: model 1 plus body mass index, smoking habit and blood pressure.

^3^Model 3: model 2 plus age.

**Fig 2:**
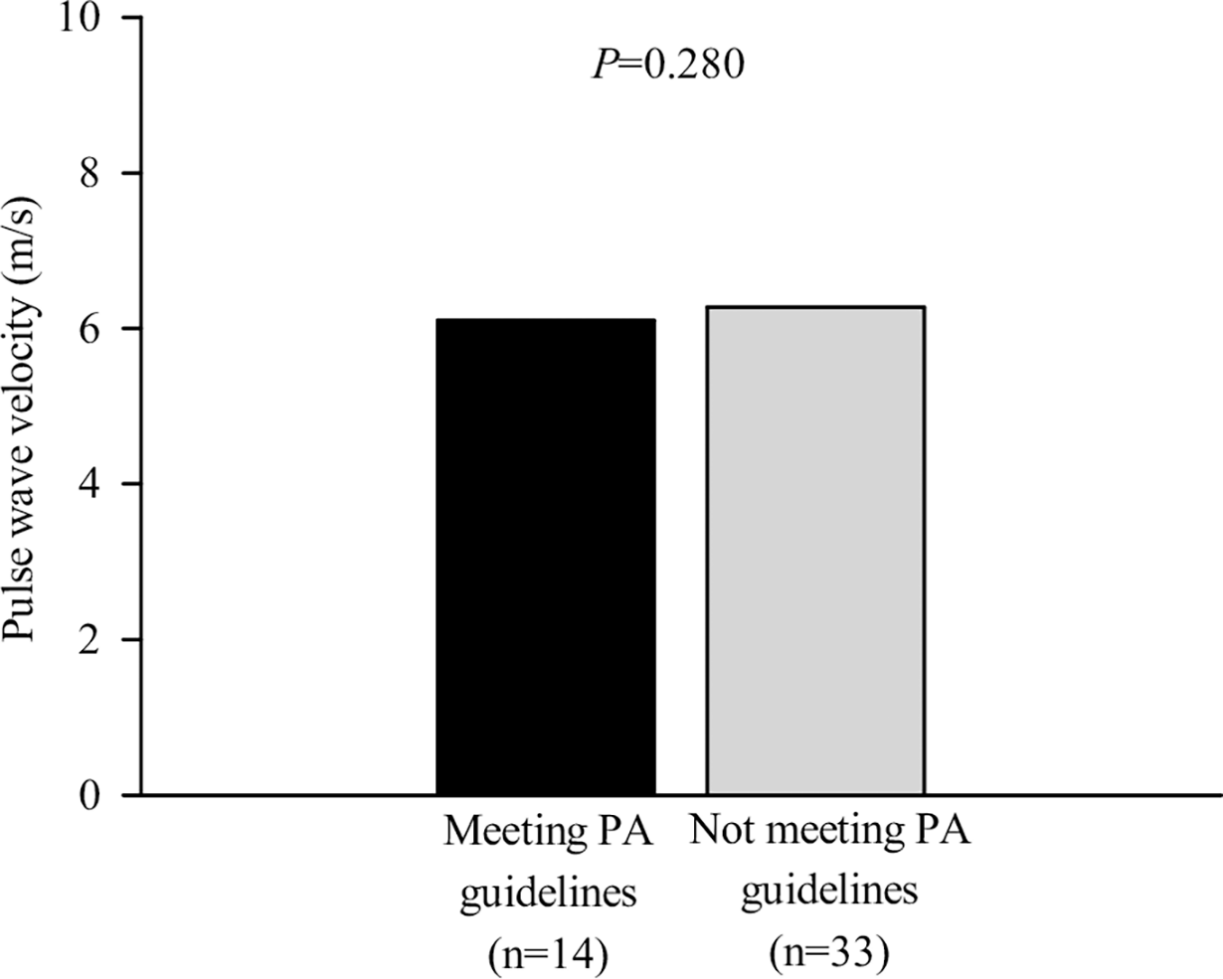
Average PWV (95% confidence interval) in women with SLE meeting and not meeting the PA guidelines.

The comparison of the average PWV between participants meeting and not meeting the PA guidelines of 150 min/week of bouted MVPA is shown in Fig 2. There were no significant differences in PWV between the two groups (b = -0.169; 95% CI: -0.480 to 0.143; *P* = 0.280).

## Discussion

The main findings of the present study indicate a lack of association of PA intensity levels and sedentary time with arterial stiffness in women with SLE. Participants who met the international PA guidelines did not show lower PWV than those not meeting them.

Benefits of regular PA for general health are well documented [33]. Parsons et al. showed that low levels of PA and high levels of sedentary time are associated with higher CVD risk among older people [34]. Furthermore, Matthews et al. evidenced that time spent in sedentary behaviours was positively associated with CVD incidence and mortality in general population, and participation in high levels of MVPA did not fully mitigate health risks associated with prolonged time watching television [35,36]. Although the sedentary time in our study was similar to that found in the general population and in patients with Sjögren's syndrome (451.4 min/day in average) [34,37], the time spent in PA of our participants differed from those observed in previous studies.

Several studies have analyzed the amount of MVPA performed by patients with SLE. For instance, Ahn et al. observed that patients with SLE performed 39.6 (SD 29.9) min/day of MVPA (measured by accelerometery) [38], while Mahieu et al. observed a total of 38.4 (SD 29.6) min/day of MVPA [39]. These numbers are tentative to think that in the present study, participants performed greater PA (i.e. 51.1 (SD 31.3) min/day of MVPA). However, these differences in PA levels might be explained by the use of an older version of accelerometer in previous studies [38,39], the GT3X ActiGraph instead of GT3X+, and because the vector magnitude from the triaxial accelerometry was computed using a different algorithm on a minute-by-minute basis to classify activity as MVPA by Ahn et al [38].

There is strong evidence supporting the well-documented public health guidelines promoting at least 150 min/week of moderate-vigorous leisure-time PA aimed at decreasing risks for metabolic diseases [11]. In addition, García-Hermoso et al. found that, in adults, long lasting periods of sedentary time were associated with a worse cardiometabolic risk profile and greater risk of clinical and subclinical atherosclerosis [15]. Pedersen and Febbraio confirmed that physical inactivity probably leads to an altered myokine response, which could provide a potential mechanism for the association between sedentary behaviour and many chronic diseases [40].

However, it is currently uncertain whether there is a link between the levels of PA, measured objectively by accelerometry, and subclinical atherosclerosis in women with SLE. Implementing strategies or lifestyle modifications to prevent CVD development is of major interest in this population. Previous studies have investigated the relationship between PA levels and sedentary lifestyle, measured by accelerometry, and clinical parameters in other autoimmune disease populations [37].

In SLE patients, it has been observed that more severe organ damage was associated with less PA of “low to moderate” intensity, compared to general population [14]. Volkmann et al. found that low PA levels were associated with increased subclinical atherosclerosis, but this association did not remain significant after controlling for traditional CVD risk factors [13]. Moreover, unlike our study, subclinical atherosclerosis was assessed by intima media thickness (IMT) and PA was investigated from self-reports questionnaires (the Medical Outcomes Study Short Form 36, SF-36, and the MESA Typical Week Physical Activity Survey). In addition, it is important to note that Kruse and Scheuermann evidenced that endothelial function declines with age and sedentary lifestyle, which are associated with an increased risk for CVD in general population [16].

The use of questionnaires to assess PA levels has shown significant differences in the estimation of PA in other populations compared to an objective measure such as accelerometry [41]. In SLE patients, accelerometer measures and IPAQ energy expenditure estimates showed a moderate agreement since individuals tend to underestimate daily walking distance, overestimate energy expenditure and overestimate time spent in MVPA with patient-reported PA instruments [38]. Despite accelerometers did not capture PA time spent in water activities, a review of the activity logs showed that this time was very low.

To the best of our knowledge, our study is the first to examine the association of objectively measured PA levels and sedentary time with PWV in a cohort of women with SLE. Based on previous literature in the general population [15,34–36], it was hypothesized that PA levels (inversely) and sedentary time (directly) would be associated with arterial stiffness. However, contrary to our hypothesis, the results of the study showed lack of association of either PA intensity levels or sedentary time with arterial stiffness. These negative results could be due to the fact that daily PA might not be sufficient to produce changes in PWV and it is necessary to perform supervised exercise training to reduce arterial distensibility in this population.

This study has limitations that might partially explain the unexpected results. The cross-sectional nature of the present study precludes establishment of causality. The participants in our study showed higher PA levels than those observed in previous studies [38,39]. Different accelerometer algorithms and/or cutoff points to determine PA intensity levels could explain these differences [18]. However, it is also possible that our sample was more physically active than previous ones, as we required clinical stability during the previous 6 months to be included in the study, just like the low average of SLEDAI and SDI showed. Furthermore, the women meeting the PA guidelines were older and presented a higher prevalence of comorbidities (such as dyslipidemia) than those not meeting the guidelines. This might be due to a higher degree of motivation to move, or a more intensive and strict cardiovascular recommendations by their physicians, which might have influenced our results. The relatively small sample size *(n = 47)* yielded a power to detect a significant association of MVPA with PWV of ~45%. Therefore, these results must be understood as preliminary/exploratory until future research with larger sample sizes confirms or contrasts our findings. Finally, the low levels of vigorous PA observed in the participants (i.e. 2.5 min/day) did not allow assessing its potential influence on PWV.

## Conclusions

Our results suggest that PA intensity levels and sedentary time are not associated with arterial stiffness in patients with SLE. Moreover, patients with SLE meeting international PA guidelines did not show lower arterial stiffness than those not meeting the PA guidelines. Future prospective research is needed to better understand the association of PA and sedentary time with arterial stiffness in patients with SLE.

## Future research

Future studies should characterize the association of physical fitness with arterial stiffness in patients with SLE. Preliminary research from our group suggests that higher cardiorespiratory fitness could attenuate the age-related arterial stiffness in women with SLE [42]. Clinical trials should also address the extent to which meeting the minimum amount of aerobic exercise might positively influence arterial stiffness this population.

## Supporting information

S1 File Database. (XLSX)
